# The Vital Statistics of Sheffield

**Published:** 1844-04

**Authors:** 


Art. VI.—*The Vital Statistics of Sheffield.* By G. Calvert Holland, Esq. m.d., Physician extraordinary to the Sheffield General Infirmary, &c.—*London,* 1843. 8vo, pp. 262.

This book is named from the contents of its eighth chapter; it is in reality a statistical history of Sheffield. The first chapter commences, like most works on local topography, with a general description of the town and neighbourhood, and notices the four rivers thereto appertaining, the geology, the men of genius, the noblemen in the neighbourhood, the water company, the botany of the district, the ornithology, the quadrupeds, the fishes in the rivers. The second chapter is headed "rate of increase in the population," but discusses, in fact, its social condition and economical progress. The third chapter is headed "comparison between the present and past periods of manufacturing distress." The fourth chapter is an inquiry into the causes of unoccupied houses; and the fifth, a comparison of the cottage accommodation in Sheffield with that in other manufacturing towns. Dr. Holland discusses also the sewerage and drainage, the streets and roads of the town, and the expenses of the highways. The eighth chapter, headed "physical condition of the population," contains the vital statistics proper of the town. In subsequent chapters, we have the statistics of the savings bank, of crime, of the well-known manufacture of Sheffield, the silver and silver-plated manufactures, and the saw, edge-tool, spring-knife, file, and fork trades. Next come the statistics of friendly societies and secret orders, of education and religious instruction, of the mechanics' institute, the literary and philosophical society, and the school of design, of the medical charitable institutions, the licensed victuallers and beerhouse keepers, and the town-trust.

Our readers will see that Dr. Holland's subject is comprehensive enough to comprehend a great deal more than "the vital statistics of Sheffield;" but, in addition to the preceding, we have politico-economical discussions, and opinions are stated and views advocated respecting which there is certainly a considerable difference of opinion. Those who take an interest in statistical researches will readily appreciate Dr. Holland's great industry and zeal, and will find the work to be a valuable addition to their statistical library; it will doubtless also become a standard book of reference for his fellow-townsmen, as it certainly merits to be. There is no want of churches or chapels at Sheffield, there being sitting-room for nearly five elevenths of the population, while not one family in twenty is in the practice of attending either at one or the other.

